# Supplementary material for: Content-rich biological network constructed by mining PubMed abstracts
Source: BMC Bioinformatics. 2004 Oct 8;5:147. doi: 10.1186/1471-2105-5-147 (PMC528731; doi:10.1186/1471-2105-5-147)
Supplement: Additional File 2 — The original results of the above study (non-essential files are deleted to keep the file size under the limit set by BMC bioinformatics). [file 1471-2105-5-147-S2.bz2 › chilibotAdditionalFile2/dip30/219ID7617032E728/html/IFNGR1_IFNG.html]

 


 **IFNGR1** and **IFNG** 
  
Found 72 abstracts in PubMed, retrieved 30.  
 

 What does Google say? 
 PDF only 
| .edu only 

---

**Interactive relationship** (e.g. stimulation, inhibition, etc)

**Neutral relationship**- Thus, membrane lipid microdomains play an important role in IFN gamma  [ **IFNG** ]  initiated endocytic events involving IFNGR 1  [ **IFNGR1** ] , and the nuclear translocation of IFN gamma  [ **IFNG** ] , IFNGR 1  [ **IFNGR1** ] , and STAT1alpha.  Ref: 12165521 J Immunol, 2002
- However, treatment of cells with IFN gamma  [ **IFNG** ]  resulted in rapid migration of IFNGR 1  [ **IFNGR1** ] , but not IFNGR  [ **IFNGR1** ]  2, from these microdomains.  Ref: 12165521 J Immunol, 2002
- A multipoint LOD score of 3.1 was obtained at  **IFNGR1** , the gene that encodes chain 1 of the interferon gamma  [ **IFNG** ]  IFN gamma  [ **IFNG** ]  receptor.  Ref: 12516030 Am J Hum Genet, 2003
- IFN gamma  [ **IFNG** ]  contains a nuclear localization sequence that may play a role in the nuclear transport of activated STAT1alpha via a complex of IFN gamma  [ **IFNG** ]  IFN gamma  [ **IFNG** ]  receptor IFNGR 1  [ **IFNGR1** ]  STAT1alpha with the nuclear importer nucleoprotein interactor 1.  Ref: 12165521 J Immunol, 2002
- Isolation of lipid microdomains from Jurkat cells showed that both IFNGR 1  [ **IFNGR1** ]  and IFNGR  [ **IFNGR1** ]  2 were associated with lipid microdomains only after stimulation with IFN gamma  [ **IFNG** ] , suggesting that the IFNGR  [ **IFNGR1** ]  subunits are recruited to lipid microdomains by IFN gamma  [ **IFNG** ]  binding in lymphocytes Jurkat in contrast to their constitutive presence in epithelial WISH cells.  Ref: 12165521 J Immunol, 2002

**Non-interactive relationship** (e.g. studied together, co-existance, homology, etc.)

- Cross phosphorylation of  **IFNGR1**  is not required for the IFN gamma  [ **IFNG** ]  like response through the chimeric receptors, nor does it mediate an IFN gamma  [ **IFNG** ]  like response to IL 6 or OSM.  Ref: 11574475 EMBO J, 2001
- These lately discovered genes, relevant to immune disorders of mononuclear phagocytes and neutrophils, include defects in the interferon gamma  [ **IFNG** ]   **IFNg**  interleukin 12 IL 12 pathway, such as  **IFNg**  receptor IFNgR  [ **IFNGR1** ]  defects, IL 12 defect, IL 12 receptor IL 12R defect, and signal transducer and activator of transcription 1 STAT 1 defect.  Ref: 12165204 Curr Allergy Asthma Rep, 2002
- Interleukin 12 receptor beta 1 IL12RB1, interleukin 12 receptor beta 2 IL12RB2, and interferon gamma  [ **IFNG** ]  receptor 1  [ **IFNGR1** ]   **IFNGR1**  perform important roles in the host defense against intracellular pathogens such as Mycobacteria.  Ref: 12743658 Immunogenetics< MedlineTA>Immunogenetics, 2002
- Complete interferon gamma  [ **IFNG** ]  receptor 1  [ **IFNGR1** ]  IFNgammaR1 deficiency is a primary immunodeficiency disease characterized by high susceptibility to recurrent, severe mycobacterial and other intracellular infections.  Ref: 12393576 Blood, 2002
- Furthermore, the numbers of SEB induced CD80 monocytes were partially decreased by anti CD119  [ **IFNGR1** ]  IFN gamma  [ **IFNG** ]  receptor MAb and by anti CD95L NOK2 MAb.  Ref: 11349026 Infect Immun, 2001
- Identification of a phorbol ester responsive element in the interferon gamma  [ **IFNG** ]  receptor 1  [ **IFNGR1** ]  chain gene.  Ref: 11477089 J Biol Chem, 2001
- Genetic basis of patients with bacille Calmette Guérin osteomyelitis in Japan identification of dominant partial interferon gamma  [ **IFNG** ]  receptor 1  [ **IFNGR1** ]  deficiency as a predominant type.  Ref: 11865431 J Infect Dis, 2002
- Reporter gene analyses of 5 deletion constructs of the IFN gamma  [ **IFNG** ]  receptor 1 gene  **IFNGR1**  promoter indicated that the critical region for control of transcription and the TPA responsive element TRE were present in the 128 to 109 base pair bp region.  Ref: 11477089 J Biol Chem, 2001
- Interferon gamma  [ **IFNG** ]  receptor 1  [ **IFNGR1** ]  IFNgammaR1 deficiency is a rare inherited immunodeficiency.  Ref: 12805054 Blood< MedlineTA>Blood, 2001
- METHODS We investigated the association of  **IFNG**  CA repeat polymorphism within the first intron, IRF1 GT repeat polymorphism within the intron 7,  **IFNGR1**  Val 14 Met, and IFNGR2 Gln 64 Arg gene polymorphisms with atopic asthma in the Japanese child population.  Ref: 11240951 J Allergy Clin Immunol, 2001
- Patients with a dominant small deletion 818del4, hotspot in the interferon gamma  [ **IFNG** ]  receptor 1  [ **IFNGR1** ]   **IFNGR1**  gene 6q23 q24 and increased susceptibility to mycobacterial infections have been recently reported.  Ref: 11781064 Clin Immunol, 2002
- Using an intronic CA n polymorphic microsatellite marker within the IFN gamma  [ **IFNG** ]  receptor 1  **IFNGR1**  gene, we have compared the allelic frequencies of this marker in hospitalized tuberculosis patients n = 120 with that of controls n = 87 from Rijeka, Croatia.  Ref: 12753505 Scand J Immunol, 2003
- Among the 12 down regulated genes, interferon gamma  [ **IFNG** ]  receptor 1  [ **IFNGR1** ]  was most notable x1 27.3.  Ref: 12823984 Exp Eye Res, 2003
- We identified a novel heterozygous single nucleotide substitution 1400 T right curved arrow C Leu 467 Pro in the seventh exon of the interferon gamma  [ **IFNG** ]  receptor 1  [ **IFNGR1** ]   **IFNGR1**  gene.  Ref: 12851715 Int J Mol Med, 2003
- Since interferon gamma  [ **IFNG** ]  is the main cytokine responsible for defence against leishmanial parasites, it was thought possible that effective antileishmanial drugs may up regulate  **IFNGR1** .  Ref: 12803856 Ann Trop Med Parasitol, 2003
- In contrast, treatments that block clathrin dependent endocytosis did not inhibit either activation or nuclear translocation of STAT1alpha or the nuclear translocation of IFN gamma  [ **IFNG** ]  or IFNGR 1  [ **IFNGR1** ] .  Ref: 12165521 J Immunol, 2002
- A novel single nucleotide substitution, Leu 467 Pro, in the interferon gamma  [ **IFNG** ]  receptor 1  [ **IFNGR1** ]  gene associated with allergic diseases.  Ref: 12851715 Int J Mol Med, 2003
- 561del4 defines a novel small deletion hotspot in the interferon gamma  [ **IFNG** ]  receptor 1  [ **IFNGR1** ]  chain.  Ref: 11781064 Clin Immunol, 2002
- In contrast, IFN gamma  [ **IFNG** ]  receptor IFNGR  [ **IFNGR1** ]  expression was increased in the presence of anti CD2 mAb.  Ref: 12744772 J Interferon Cytokine Res, 2003
- By Western blotting the plasma membrane raft fractions obtained using either non detergent or detergent based methods contained significant amounts of STAT1 and STAT3 up to approximately 10% of the total cytoplasmic amount as well as the integral raft proteins caveolin 1 and flotillin 1, the IL 6 receptor signal transducing chain gp130, the interferon gamma  [ **IFNG** ]  receptor alpha chain  [ **IFNGR1** ]  IFN gammaRalpha, and the chaperone glucose regulated protein 58 GRP58 ER 60 ERp57.  Ref: 11815625 J Biol Chem, 2002
- This study explored the relationship between polymorphisms in the promoter region of the gene encoding IFN gamma  [ **IFNG** ]  receptor 1  **IFNGR1**  and susceptibility to malaria in African children.  Ref: 12023780 J Infect Dis, 2002
- Different types of mutations in four genes  **IFNGR1** , IFNGR2, IL12B, IL12RB1 have revealed both allelic and nonallelic heterogeneity and result in eight different disorders whose common pathogenic pathway is impaired interferon gamma  [ **IFNG** ]  IFNgamma mediated immunity.  Ref: 11420412 Pediatr Res, 2001
- Filipin pretreatment, which specifically inhibits endocytosis from caveolae like microdomains, inhibited the nuclear translocation of IFN gamma  [ **IFNG** ]  and IFNGR 1  [ **IFNGR1** ]  as well as the tyrosine phosphorylation and nuclear translocation of STAT1alpha, but did not affect the binding of IFN gamma  [ **IFNG** ]  to these cells.  Ref: 12165521 J Immunol, 2002
- Interferon gamma  [ **IFNG** ]  receptor 1  [ **IFNGR1** ]  gene polymorphism in tuberculosis patients from Croatia.  Ref: 12753505 Scand J Immunol, 2003
- The  **IFNGR1**  signal transducing subunit of the IFN gamma  [ **IFNG** ]  receptor is tyrosine phosphorylated through the chimeric receptors and the endogenous IL 6 and OSM receptors.  Ref: 11574475 EMBO J, 2001
- We previously showed decreased OPN expression in the granulomas of patients with disseminated mycobacterial infection in the setting of mutation of the IFN gamma  [ **IFNG** ]  receptor 1 IFNGR 1  [ **IFNGR1** ] .  Ref: 12804068 J Interferon Cytokine Res, 2003
- Correction of complete interferon gamma  [ **IFNG** ]  receptor 1  [ **IFNGR1** ]  deficiency by bone marrow transplantation.  Ref: 12393576 Blood, 2002
- Modulation and implications in pathogenesis of interferon gamma  [ **IFNG** ]  receptor 1  [ **IFNGR1** ]  in patients with chronic hepatitis B < ArticleTitle>.  Ref: 12546733 Zhonghua Gan Zang Bing Za Zhi, 2003
- Missense mutations of the interleukin 12 receptor beta 1 IL12RB1 and interferon gamma  [ **IFNG** ]  receptor 1  [ **IFNGR1** ]   **IFNGR1**  genes are not associated with susceptibility to lepromatous leprosy in Korea.  Ref: 12743658 Immunogenetics< MedlineTA>Immunogenetics, 2003
- Persistent Mycobacterium avium infection following non myeloablative allogeneic peripheral blood stem cell transplantation for Interferon gamma  [ **IFNG** ]  receptor 1  [ **IFNGR1** ]  deficiency.  Ref: 12805054 Blood< MedlineTA>Blood, 2003
- Apparently for the first time, the peripheral blood monocytes of individuals with active visceral leishmaniasis VL have been found to show reduced expression of interferon gamma  [ **IFNG** ]  receptor 1  [ **IFNGR1** ]   **IFNGR1** .  Ref: 12803856 Ann Trop Med Parasitol, 2003
- Recently, recessive mutations in the interferon gamma  [ **IFNG** ]  receptor ligand binding chain  **IFNgR1** , interferon gamma  [ **IFNG** ]  receptor signalling chain IFNgR2, interleukin 12 p40 subunit IL 12p40, and interleukin 12 receptor beta 1 chain IL 12Rb1 genes have been identified in a number of patients with disseminated BCG or NTM infection.  Ref: 11261247 Bull Acad Natl Med, 2000
- Antileishmanial drugs cause up regulation of interferon gamma  [ **IFNG** ]  receptor 1  [ **IFNGR1** ] , not only in the monocytes of visceral leishmaniasis cases but also in cultured THP1 cells.  Ref: 12803856 Ann Trop Med Parasitol, 2003
- Both surface and intracellular staining of all cell lines with antibodies against  **IFNgR1**  and IFNgR2 failed to detect any increase in  **IFNg**  receptor expression following incubation with PMA.  Ref: 12787470 Cancer Cell Int< MedlineTA>Cancer Cell Int, 2003
